# Interpretable machine learning for preoperative prediction of WHO/ISUP grade in clear cell renal cell carcinoma using contrast-enhanced CT–derived extracellular volume fraction

**DOI:** 10.3389/fonc.2026.1851636

**Published:** 2026-10-13

**Authors:** Yating Wang, Ying Wang, Genji Bai, Wei Huang, Min Huang, Wei Chen

**Affiliations:** 1 Department of medical imaging, Affiliated Huaian No.1 People’s Hospital of Nanjing Medical University, Huai’an, China; 2 Department of Medical Imaging, Siyang First People’s Hospital, Suqian, China

**Keywords:** clear cell renal cell carcinoma, computed tomography, extracellular volume fraction, machine learning, WHO/ISUP grading

## Abstract

**Objective:**

To develop and validate an interpretable machine learning (ML) model that integrates extracellular volume fraction (ECV) derived from contrast-enhanced CT with clinical and imaging features for preoperative prediction of World Health Organization/International Society of Urological Pathology (WHO/ISUP) grade in clear cell renal cell carcinoma (ccRCC).

**Materials and methods:**

A retrospective cohort of 263 patients with pathologically confirmed ccRCC from two institutions was analyzed. Patients from Hospital A (n=184) were randomly divided into training (n=128) and internal validation (n=56) cohorts, while patients from Hospital B (n=79) served as an independent external validation cohort. CT-derived ECV, arterial enhancement fraction (AEF), and conventional imaging features were extracted. Six machine learning models (Logistic Regression [LR], eXtreme Gradient Boosting [XGBoost], Gaussian Naive Bayes [GNB], Support Vector Machine [SVM], K-Nearest Neighbors [KNN], Light Gradient Boosting Machine [LightGBM]) were constructed, and the best-performing model was identified based on bootstrap resampling. Model interpretability was assessed using SHapley Additive exPlanations (SHAP).

**Results:**

Tumor size (low-grade: 4.99 ± 1.98 cm; high-grade: 6.20 ± 2.72 cm, P = 0.002), ECV (low-grade: 0.46 ± 0.09; high-grade: 0.57 ± 0.12, P<0.001), and AEF (low-grade: 1.37 ± 0.24; high-grade: 1.56 ± 0.25, P<0.001) were significantly higher in high-grade ccRCC, while hematocrit (HCT, low-grade: 0.41 ± 0.05; high-grade: 0.38 ± 0.06, P<0.001) was lower. The optimal LR model achieved robust performance: training cohort AUC = 0.995 (95% CI: 0.990–0.999), internal validation AUC = 0.903 (95% CI: 0.818–0.988), external test AUC = 0.857 (95% CI: 0.756–0.958). SHAP analysis showed ECV, tumor diameter, and AEF were the top 3 predictors.

**Conclusion:**

An interpretable ML model incorporating CT-derived ECV and imaging features effectively predicts preoperative WHO/ISUP grade of ccRCC, providing a noninvasive tool for individualized risk stratification and clinical decision-making.

## 1 Introduction

Clear cell renal cell carcinoma (ccRCC) is the most common subtype of renal malignancy in adults, characterized by highly heterogeneous biological behavior and thus variable clinical outcomes (1, 2). According to the World Health Organization/International Society of Urological Pathology (WHO/ISUP) grading system, tumor grade is a well-established prognostic factor that reflects tumor aggressiveness and patient outcomes (3). Accurate preoperative prediction of ccRCC grade is therefore of great clinical importance, as it can assist in tailoring surgical strategies and individualized follow-up management. However, grading currently relies primarily on postoperative histopathological examination, and effective noninvasive approaches remain limited (4).

To address this limitation, the advancement of medical imaging has led to the widespread use of contrast-enhanced computed tomography (CT) in the diagnosis and assessment of renal tumors. Extracellular volume fraction (ECV), a quantitative imaging biomarker derived from enhanced CT, has recently attracted increasing attention (5, 6). ECV reflects extracellular matrix composition and tumor microenvironmental features, and prior studies have shown its close association with tumor cellularity, angiogenesis, and stromal content (7). These findings suggest that ECV may serve as a promising imaging biomarker to characterize the biological behavior of ccRCC. Nonetheless, the predictive ability of a single imaging feature is often limited, as it may not capture the full complexity of tumor heterogeneity, thereby restricting its clinical applicability.

Machine learning (ML) techniques have shown great promise in radiomics and predictive modeling (8, 9). By integrating multidimensional clinical, imaging, and quantitative parameters, ML algorithms can uncover hidden relationships within complex datasets and provide more accurate predictive tools for tumor grading (10). However, existing studies often suffer from several limitations: small sample sizes that hinder model robustness and generalizability; insufficient integration of quantitative imaging biomarkers such as ECV; and reliance on “black-box” models with limited interpretability, raising concerns about clinical translation.

The aim of this study was therefore to develop an interpretable ML model for preoperative prediction of ccRCC WHO/ISUP grade, integrating CT-derived ECV with clinical and imaging features. By applying SHapley Additive exPlanations (SHAP) analysis, we sought to elucidate the contribution of individual predictors, enhancing model transparency and clinical applicability.

## 2 Materials and methods

### 2.1 General information

221 patients with renal tumors were retrospectively collected between January 2017 and December 2024. All patients underwent surgical treatment, and resected tumor specimens were subjected to histopathological analysis. Baseline clinical characteristics potentially associated with tumor grading (e.g., age, sex, tumor size, hematocrit [HCT]) were retrieved from the hospital electronic medical record system. Inclusion criteria: (1) Histopathologically and immunohistochemically confirmed ccRCC with definitive WHO/ISUP grading; (2) Multiphase contrast-enhanced CT examination performed within 2 weeks before surgery, with adequate image quality for analysis; (3) Complete baseline clinical information. Exclusion criteria: (1) Prior chemotherapy or radiotherapy (n=11); (2) Poor CT image quality due to motion artifacts or contrast agent injection issues (n=14); (3) Incomplete imaging data (n=12). Ultimately, 184 ccRCC patients from Hospital A were included and randomly divided into training (n=128) and internal validation (n=56) cohorts at a 7:3 ratio. An independent external validation cohort comprising 79 ccRCC patients from Siyang People’s Hospital (Hospital B) was included to assess model generalizability. The patient enrollment process is shown in **Figure 1**.

**Figure 1:**
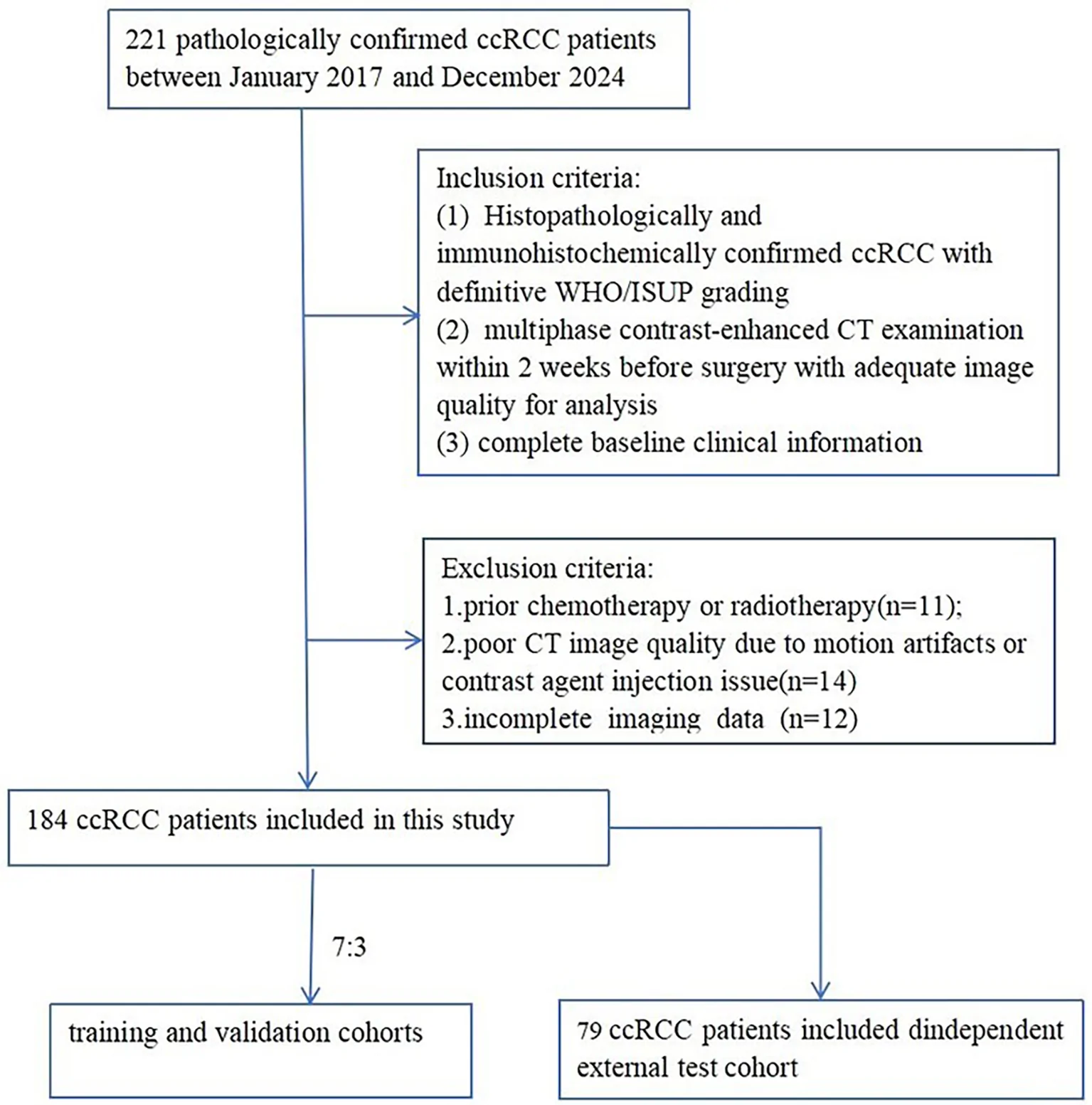
Patient enrollment flowchart.

### 2.2 Pathological analysis

Histopathological evaluation was performed using hematoxylin–eosin (HE) staining and immunohistochemistry (IHC) techniques. Two pathologists with more than 10 years of clinical and pathological experience independently assessed tumor grading in a double-blind manner, in strict accordance with the 2016 WHO/ISUP classification system (11). Discrepancies were resolved through joint discussion. Tumors were categorized into two groups: low-grade (WHO/ISUP grades 1–2) and high-grade (WHO/ISUP grades 3–4).

### 2.3 CT examination protocol

Multiphase dynamic contrast-enhanced CT (MDCT) imaging was acquired using three different CT scanners, including SOMATOM Definition Flash (Siemens Healthineers), Discovery 750 (GE Healthcare), and SOMATOM go.top (Siemens Healthineers). Detailed scanning and reconstruction parameters are summarized in **Table 1**.

**Table 1:** CT examination technique.

Parameter	SOMATOM Definition Flash	Discovery 750	SOMATOM go.top
Trigger attenuation threshold (Hu)	120	150	150
CMP (s)	30-35	30-35	25-35
NP (s)	80-90	80-90	50-60
DP (min)	5-8	5-10	5-8
Tube voltage (kV)	100-120	100-120	100-120
Reference tube current (mAs)	150-180	150-180	150-180
Collimation (mm)	80	80	80
Pitch	1	1	0.992
Rotation time (s)	0.5	0.5	0.5
Reformatted section thickness (mm)	0.625	0.625	1.25
Reformatted section increment (mm)	1	1	1

CMP, Corticomedullary phase; NP, Nephrographic phase; DP, Delayed phase

### 2.4 CT image analysis

#### 2.4.1 Image preprocessing

All CT images were resampled to 1×1×3 mm isotropic voxels to unify disparate slice thicknesses. Linear gray-scale HU normalization was subsequently applied to unenhanced, corticomedullary and delayed-phase images to correct baseline HU variations arising from variable tube currents and scanner hardware. These standardized preprocessing pipelines partially mitigate equipment-related bias in ECV and AEF quantification.

#### 2.4.2 Imaging features

Conventional CT features included tumor size, intratumoral calcification, necrosis/cystic degeneration, intratumoral vessels, renal vein thrombus, collecting system invasion, lymph node metastasis, and enhancement pattern. Two radiologists independently assessed these features, with consensus reached through discussion.

#### 2.4.3 Calculation of ECV and AEF

Regions of interest (ROIs) were placed on the most intensely enhanced solid regions of the tumor during the nephrographic phase, avoiding necrotic, cystic, calcified, or hemorrhagic areas. ROIs were matched across unenhanced and enhanced phases. At the level of the ipsilateral renal artery origin, aortic ROIs (25–100 mm²) were selected. All ROIs were manually drawn as two-dimensional single-slice regions on the nephrographic-phase image showing the maximum solid cross-section of the tumor, rather than volumetric whole-tumor ROIs. CT attenuation values were measured three times during unenhanced and delayed phases for both tumor and aorta, and the mean was recorded. HCT values were retrieved from routine blood tests within 24 hours. Inter-observer reproducibility for manual ROI placement was assessed. Two board-certified radiologists with 10-year imaging experience independently drew ROIs on a random subset of 80 cases (30% of the total cohort) from both Hospital A and Hospital B, blinded to each other’s segmentations and final pathological diagnoses. ROIs were positioned on the most intensely enhanced solid tumor regions following the aforementioned protocol. The intra-class correlation coefficient (ICC) was calculated for core quantitative parameters. ICC values were greater than 0.85 for ECV, AEF, and tumor diameter, demonstrating excellent inter-reader consistency and confirming the stability of manual solid-region ROI delineation. Only quantitative features with ICC > 0.75 were retained for subsequent model construction.

The ECV was calculated using the formula:


$$\text{ECV }\left(\%\right)\;=\;\left(\text{ΔH}{\text{U}}_{\text{tumor}}/\text{ΔH}{\text{U}}_{\text{aorta}}\right)\;\times \left(1-\text{HCT}\right)\;\times 100\%$$

Where ΔHUtumor and ΔHUaorta denote the differences between CT values in the delayed phase and the non-enhancement phase (NEP) for the solid component of the tumor and the abdominal aorta, respectively.

Arterial enhancement fraction (AEF) was calculated using the formula:


$$\text{AEF }=\;\left(\text{ΔH}{\text{U}}_{\text{CMP}}-\text{ΔH}{\text{U}}_{\text{NEP}}\right)/\left(\text{ΔH}{\text{U}}_{\text{NP}}-\text{ΔH}{\text{U}}_{\text{NEP}}\right)\;\times 100\%$$

Where ΔHU_CMP_ = CT value in corticomedullary phase − CT value in NEP; ΔHU_NP_ = CT value in nephrographic phase − CT value in NEP.

### 2.5 Model development and evaluation

Data balancing: Due to class imbalance (fewer high-grade cases), the Synthetic Minority Oversampling Technique (SMOTE) was applied to the training cohort. (2) Data splitting: A total of 184 patients from Hospital A were split (7:3) into training and validation cohorts, while 79 patients from Hospital B served as an external validation cohort. (3) Model construction: Six machine learning models were developed, including Logistic Regression (LR), eXtreme Gradient Boosting (XGBoost), Gaussian Naïve Bayes (GNB), Support Vector Machine (SVM), K-Nearest Neighbors (KNN), and Light Gradient Boosting Machine (LightGBM). The evaluation metrics included accuracy, sensitivity, specificity, positive predictive value (PPV), negative predictive value (NPV), and F1-score. The best-performing model was selected based on bootstrap iterations. (4) Model validation: The optimal model was first trained on the training cohort, then evaluated on the validation cohort with five-fold cross-validation conducted. Additionally, independent external testing was performed on the cohort from Hospital B. (5) Model interpretation: SHAP values were calculated to quantify feature importance and visualize feature impact on predictions. The flowchart of model development and evaluation was illustrated in **Figure 2**. Hyper-parameters for each machine-learning classifier were optimized by five-fold cross-validation on the training cohort, with fixed random seeds applied for reproducibility. Full annotated analysis scripts and detailed hyperparameter settings are available from the corresponding author upon reasonable academic request.

**Figure 2:**
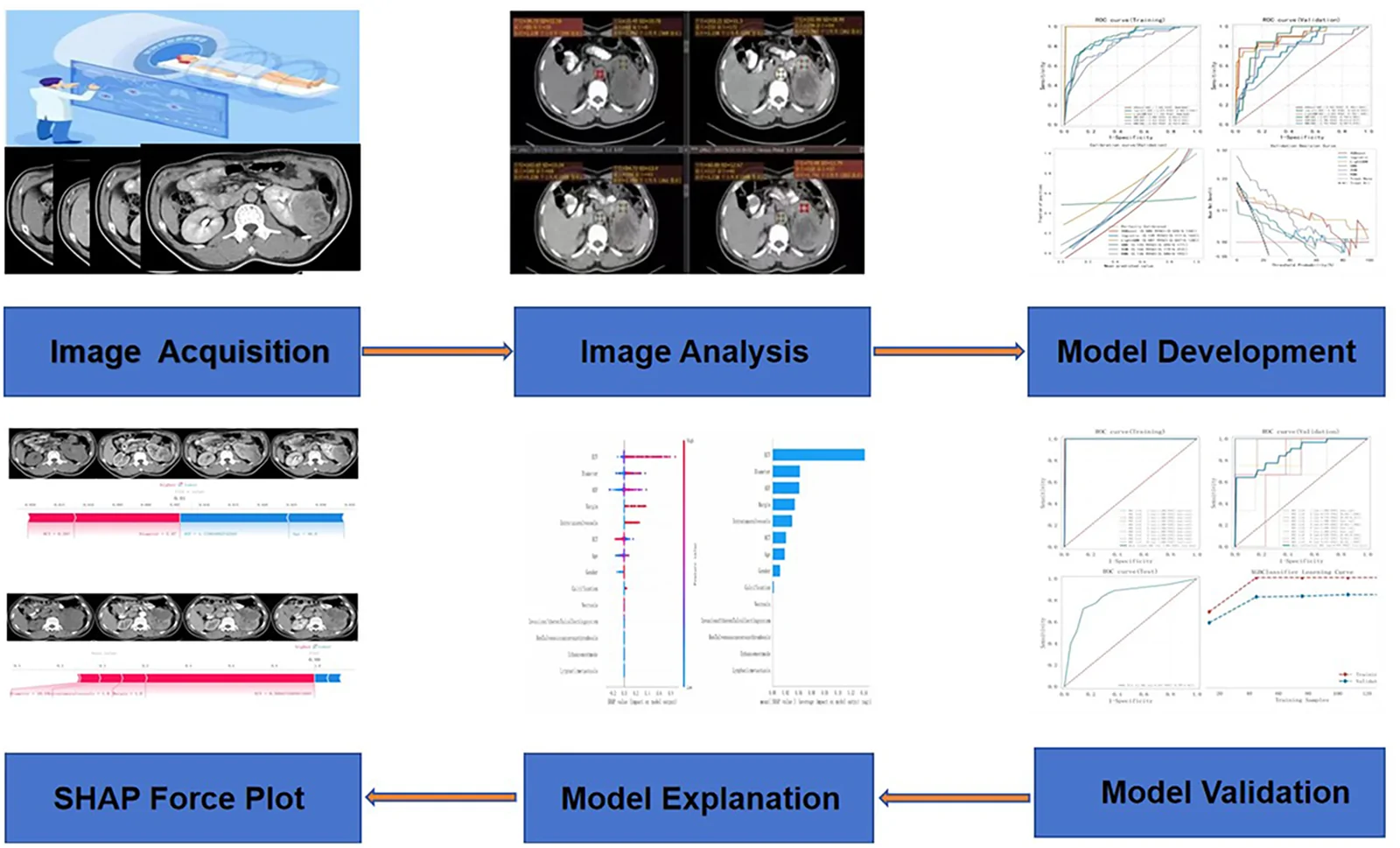
The flowchart of model development and evaluation.

### 2.6 Statistical analysis

Statistical analyses were performed using MedCalc (version 20.1) and R software (version 4.2.1). Continuous variables were tested for normality first; normally distributed data were analyzed using Student’s t-test and summarized as mean ± standard deviation (SD), while non-normally distributed data were analyzed using the Mann-Whitney U test and summarized as median ± interquartile range (IQR). Categorical variables were analyzed using the chi-square test or Fisher’s exact test. Receiver operating characteristic (ROC) curves and the corresponding area under the curve (AUC) values were used to evaluate model performance. Calibration curves assessed agreement between predicted and observed probabilities. Decision curve analysis (DCA) evaluated clinical net benefit. Sensitivity, specificity, accuracy, positive predictive value (PPV), and negative predictive value (NPV) were calculated for both training and validation cohorts. The DeLong test compared AUC values. P<0.05 was considered statistically significant.

## 3 Results

### 3.1 Clinical baseline data and CT features

This study included 263 patients with ccRCC: low-grade group (n=171, 65%) and high-grade group (n=92, 35%). For internal validation, 184 patients from Hospital A were randomly divided into an internal training (n=128, 62.5%) and internal validation cohorts (n=56, 37.5%) at a 7:3 ratio. 79 patients from Hospital B formed the external validation cohort. All clinical baseline data and CT features are presented in **Table 2**. Baseline clinical, laboratory, and CT imaging features were well balanced between the twocohorts with no significant inter-group differences (all *P* > 0.05), as validated in **Supplementary Table S1**.

**Table 2:** Patient clinical baseline data and CT features.

Feature parameter	Training and validation cohort			Test cohort		
	LG (n=115)	HG (n=69)	*P value*	LG (n=56)	HG (n=23)	*P value*
Age	58.86 ± 10.80	60.55 ± 10.40	*P* = 0.299	58.23 ± 11.07	61.04 ± 10.42	*P* = 0.291
Sex			*P* = 0.636			*P* = 0.022
Female	41	27		25	4	
Male	74	42		31	19	
HCT	0.41 ± 0.05	0.38 ± 0.06	*P*<0.001	0.42 ± 0.05	0.41 ± 0.05	*P* = 0.620
Diameter	4.99 ± 1.98	6.20 ± 2.72	*P* = 0.002	4.43 ± 1.79	4.57 ± 1.82	*P* = 0.761
AEF	1.37 ± 0.24	1.56 ± 0.25	*P*<0.001	1.18 ± 0.35	1.36 ± 0.48	*P* = 0.072
ECV	0.46 ± 0.09	0.57 ± 0.12	*P*<0.001	0.43 ± 0.12	0.61 ± 0.14	*P<*0.001
Necrosis			*P* = 0.743			*P* = 0.552
None	17	9		18	9	
Present	98	60		38	14	
Calcification			*P* = 0.463			*P* = 0.642
None	104	60		52	21	
Present	11	9		4	2	
Margin			*P*<0.001			*P* = 0.200
Clear	95	39		40	16	
Blurry	20	30		13	10	
Intratumoral vessels			*P* = 0.001			*P* = 0.995
None	91	38		39	17	
Present	24	31		16	7	
Renal vein tumor thrombus			*P*<0.001			*P* = 0.510
None	115	60		55	22	
Present	0	9		1	1	
Renal collecting system invasion			*P* = 0.007			*P* = 0.527
None	106	54		45	11	
Present	9	15		17	6	
Lymph node metastasis			*P* = 0.117			*P* = 0.258
None	114	66		55	21	
Present	1	3		1	2	
Enhancement pattern			*P* = 0.117			*P* = 0.704
Homogeneous	111	69		17	39	
Heterogeneous	4	0		6	17	

LG, Low grade; HG, High grade; AEF, Arterial enhancement fraction; ECV, Extracellular volume fraction; HCT, Hematocrit

### 3.2 Machine learning model construction and evaluation

Results of the six ML models are shown in **Table 3**. In the training cohort, AUC performance ranked: XGBoost (0.982) > LightGBM (0.977) > SVM (0.931) > LR (0.910) > KNN (0.895) > GNB (0.840). In the internal validation cohort, LR achieved the highest AUC (0.892, 95% CI: 0.775–0.999), followed by SVM (0.871, 95% CI: 0.744–0.990). XGBoost and LightGBM showed decreased AUC (0.862 and 0.859, respectively), indicating mild overfitting. DCA and calibration curves confirmed the LR had the most favorable clinical utility (net benefit >0.13 across threshold probabilities) and calibration (calibration error: 0.125, 95% CI: 0.102–0.149). Thus, LR was identified as the optimal model (**Figure 3**). We performed pairwise DeLong tests to compare the AUCs of the top-performing models in the internal validation cohort. Specifically, we compared LR (AUC = 0.892) against the next-best performing models: SVM (AUC = 0.871), XGBoost (AUC = 0.862), and LightGBM (AUC = 0.859). The DeLong test revealed that LR significantly outperformed SVM (*P*< 0.001), XGBoost (*P*< 0.001) and LightGBM (*P*< 0.001). These results provide statistical evidence supporting the selection of LR as the optimal model.

**Table 3:** Predictive performance of different machine learning models.

Dataset	Model	AUC (95% CI)	Accuracy		Sensitivity	Specificity	F1 score
Training cohort	LR	0.910 (0.859-0.961)		0.852	0.873	0.839	0.816
	XGBoost	0.982 (0.963-0.999)		0.947	0.909	0.970	0.927
	GNB	0.840 (0.775-0.906)		0.792	0.790	0.793	0.739
	SVM	0.931 (0.881-0.981)		0.890	0.880	0.896	0.858
	KNN	0.895 (0.846-0.943)		0.810	0.891	0.761	0.779
	LightGBM	0.977 (0.955-0.998)		0.944	0.928	0.954	0.779
Validation cohort	LR	0.892 (0.775-0.999)		0.809	0.784	0.826	0.755
	XGBoost	0.862 (0.734-0.990)		0.783	0.624	0.878	0.675
	GNB	0.787 (0.625-0.949)		0.739	0.738	0.739	0.686
	SVM	0.871 (0.744-0.990)		0.793	0.799	0.791	0.744
	KNN	0.784 (0.634-0.934)		0.701	0.740	0.678	0.647
	LightGBM	0.859 (0.723-0.991)		0.804	0.767	0.826	0.739

LR, Logistic Regression; XGBoost, Extreme Gradient Boosting; GNB, Gaussian Naive Bayes; SVM, Support Vector Machine; KNN, K-Nearest Neighbors; LightGBM, Light Gradient Boosting Machine; AUC, Area under curve; CI, Confidence interval

**Figure 3:**
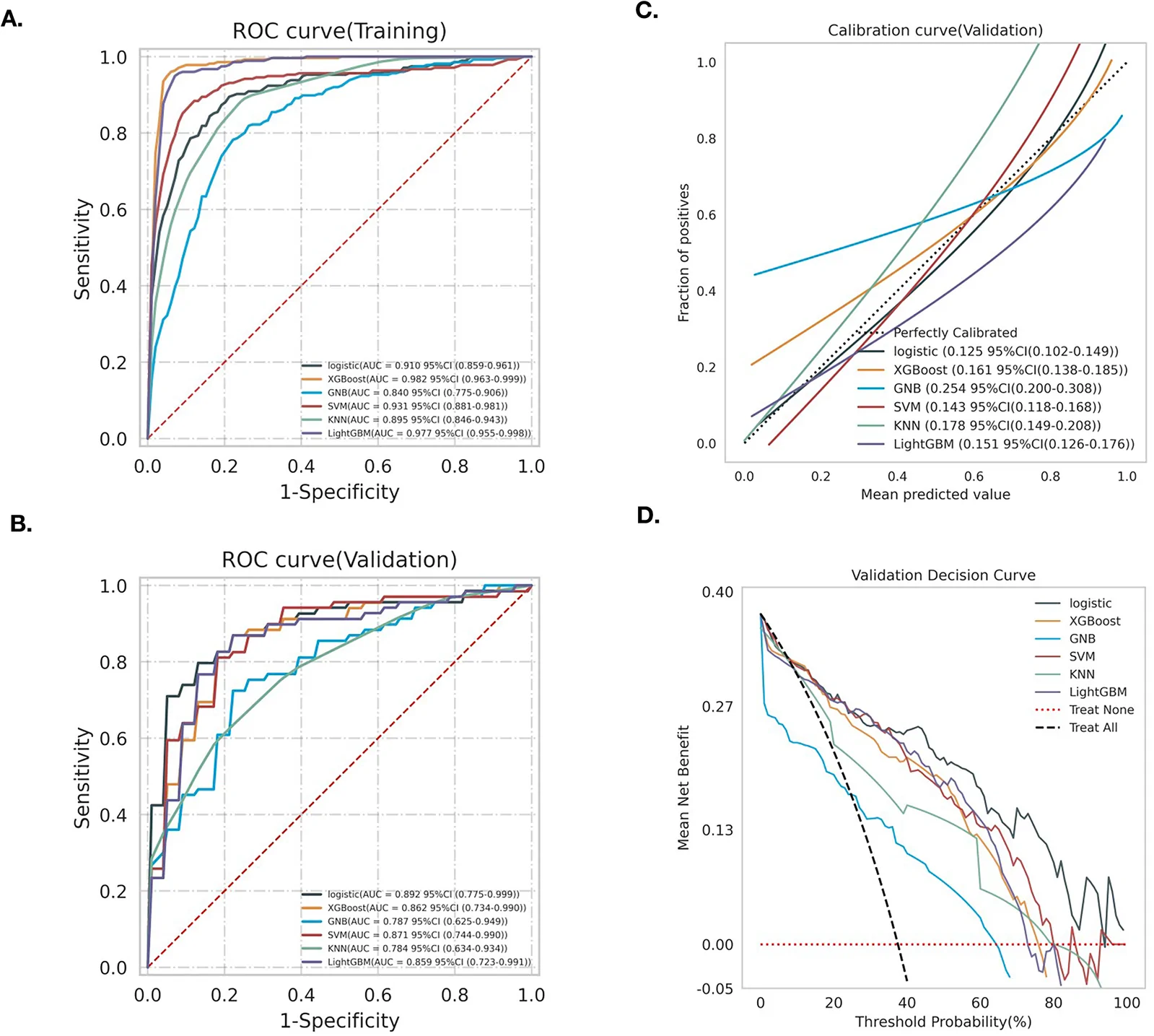
Evaluation of six machine learning models. **(A)** ROC of the models in training set. **(B)** ROC of the models in validation cohort. **(C)** Calibration curve of the models. 3D: Decision curve of the models in validation.

### 3.3 Establishment and evaluation of the optimal model

Five-fold cross-validation of the LR model showed a mean AUC of 0.995 (95% CI: 0.990–0.999) in the training cohort and 0.903 (95% CI: 0.818–0.988) in the internal validation cohort. In the external validation cohort, the LR model achieved an AUC of 0.857 (95% CI: 0.756–0.958), with accuracy=83.5%, sensitivity=60.9%, specificity=92.9%, PPV = 77.8%, NPV = 85.2%, and F1-score=68.3% (**Table 4**; **Figure 4**).

**Table 4:** Detailed performance of the optimal LR model.

Parameter	Training cohort	Validation cohort	Test cohort
AUC (95%CI)	0.995(0.990-0.999)	0.903(0.818-0.988)	0.857(0.756-0.958)
Cut-off value	0.698	0.698	0.669
Accuracy	0.971	0.806	0.835
Sensitivity	0.938	0.604	0.609
Specificity	0.991	0.925	0.929
PPV	0.984	0.830	0.778
NPV	0.964	0.811	0.852
F1 Score	0.960	0.678	0.683

AUC, Area under curve; PPV, Positive predictive value; NPV, Negative predictive value; CI, Confidence Interval

**Figure 4:**
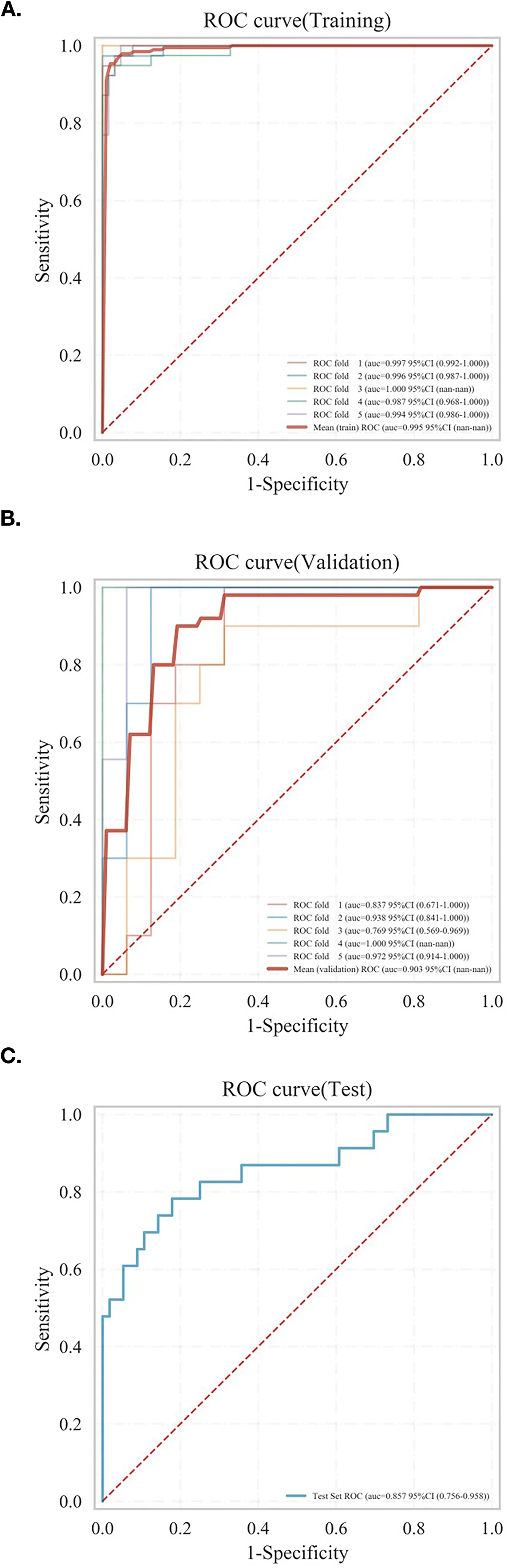
Evaluation of the optimal model. **(A)** ROC of 5-fold cross-validation in training cohort. **(B)** ROC of 5-fold cross-validation in validation cohort. **(C)** ROC of the model in test cohort.

### 3.4 Model interpretability analysis

To intuitively explain the contribution of selected variables to the prediction model, SHAP—a widely used model interpretability tool—was employed. For the overall feature analysis: the SHAP value importance bar plot (**Figure 5A**) revealed that ECV, tumor diameter, and AEF ranked as the top three most important features, with ECV exhibiting the highest importance weight. The SHAP bee swarm plot (**Figure 5B**) demonstrated that higher ECV, tumor diameter, and AEF values were associated with increased probability of high-grade ccRCC (red dots: positive impact; blue dots: negative impact). The SHAP dependence plots were further generated for the top three continuous predictors (ECV, tumor diameter, and AEF) to visualize their marginal effects on the predicted probability of high-grade ccRCC (**Figures 5C–E**). The SHAP dependence plots demonstrated that the predicted risk of high-grade ccRCC generally increased as ECV, tumor diameter and AEF rose. Distinct steep segments were observed on the curves, marking the value ranges where the predicted probability of high-grade ccRCC increased rapidly. Although these intervals cannot serve as definitive diagnostic cut-offs, they provide quantitative, clinically interpretable reference ranges for risk stratification. Individual visualization plots (**Figures 6A, B**) presented two typical examples: one correctly predicted low-grade ccRCC case and one correctly predicted high-grade ccRCC case. Additionally, SHAP attribution force plots clarified the positive and negative contributions of each feature to the prediction outcome in individual cases. Notably, the baseline value in these force plots represented the average predicted probability of the model for the entire dataset, whereas f(x) denoted the final predicted probability for the specific case in question.

**Figure 5:**
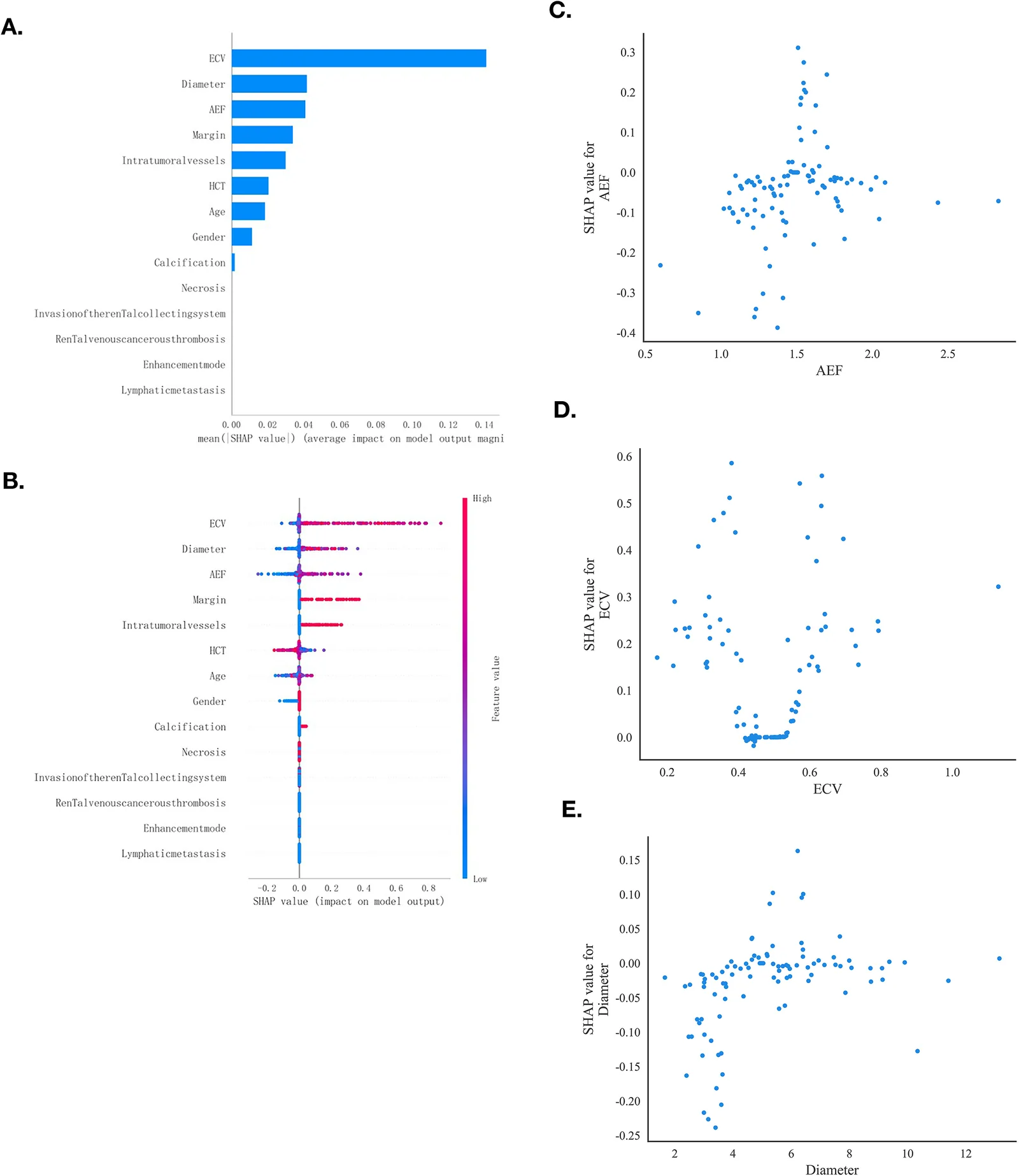
SHAP interpretation and partial dependence analysis of the predictive model for high-grade ccRCC. **(A)** SHAP feature importance bar plot showing the mean absolute SHAP values of all predictors, with ECV, tumor diameter and AEF identified as the three most influential features. **(B)** SHAP bee swarm plot illustrating the direction and magnitude of feature effects on model prediction. Higher values of ECV, tumor diameter and AEF were associated with an increased probability of high-grade ccRCC. **(C–E)** The SHAP dependence plots for ECV, tumor diameter, and AEF, respectively. The x-axis represents the raw value of each continuous featurFor patients predicted to have low-grade lesione, and the y-axis denotes the model-predicted probability of high-grade ccRCC. The curves depict marginal changes in predicted risk across the full range of feature values, highlighting value intervals where the risk of high-grade ccRCC rises sharply.

**Figure 6:**
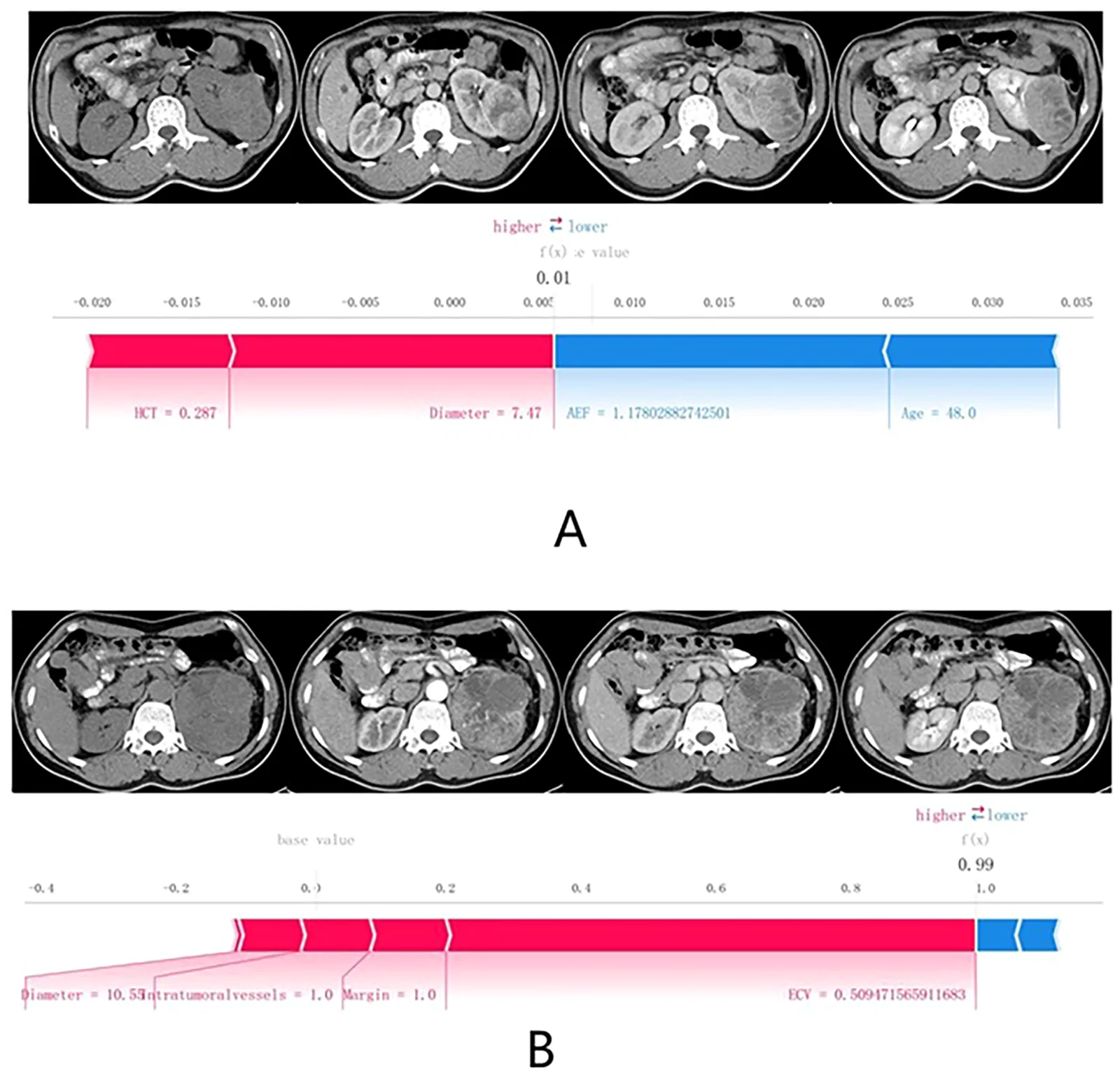
Two representative examples of accurate predictions **(A)** Individual features of patients with high-grade ccRCC. **(B)** Individual features of patients with low-grade ccRCC. The horizontal axis shows the cumulative impact of each feature on the predicted probability (f(x)). The baseline value (gray vertical line) represents the average predicted probability of high-grade ccRCC across the entire dataset. Features with positive values push the prediction toward high-grade, while negative values pull it toward low-grade.

## 4 Discussion

In this study, we developed and validated an interpretable machine learning (ML) model based on contrast-enhanced CT-derived extracellular volume fraction (ECV), combined with clinical and imaging features, for preoperative prediction of WHO/ISUP grade in clear cell renal cell carcinoma (ccRCC). The model achieved robust performance across training (AUC = 0.995), internal validation (AUC = 0.903), and external test (AUC = 0.857) cohorts, highlighting ECV’s potential as a noninvasive imaging biomarker and the value of interpretable ML in ccRCC risk stratification.

ECV—a quantitative parameter reflecting extracellular matrix composition, vascularity, and tumor cell density—has been linked to aggressive tumor behavior in multiple malignancies (12–15). Consistent with prior studies (13), our study demonstrated that ECV was among the most important predictors for distinguishing between low-and high-grade ccRCC. Mechanistically, the grading heterogeneity of ccRCC is fundamentally driven by aberrant molecular signaling and progressive remodeling of the tumor microenvironment (TME), which provides the intrinsic pathological basis for our imaging-based predictive model. Von hippel-lidau (VHL) gene inactivation is the hallmark genetic alteration of ccRCC, which leads to persistent accumulation of hypoxia-inducible factor (HIF-1α/2α) under normoxic conditions and subsequent overactivation of downstream pro-angiogenic pathways, particularly vascular endothelial growth factor (VEGF) signaling (3, 10). Continuous VEGF overexpression drives excessive, disorganized angiogenesis, forming tortuous, hyperpermeable microvessels with structural defects, which significantly increase tumor blood perfusion and contrast agent extravasation (1, 7). Higher-grade ccRCC exhibits more pronounced HIF-VEGF axis activation, accompanied by increased cancer-associated fibroblast infiltration, stromal hyperplasia, and extracellular matrix remodeling, further expanding the tumor extracellular space (2, 5). Collectively, these progressive pathological changes across different tumor grades directly alter contrast-enhanced CT perfusion and distribution characteristics, enabling quantitative differentiation via ECV and AEF. As objective quantitative imaging biomarkers, ECV and AEF precisely reflect the above grade-dependent TME alterations from multiple dimensions. ECV quantitatively quantifies the proportion of tumor extracellular space, which positively correlates with stromal hyperplasia, microvascular permeability, and tumor aggressiveness (13). High-grade ccRCC with severe vascular leakage and stromal expansion inevitably presents significantly elevated ECV values, which is consistent with our statistical results that high-grade lesions had markedly higher ECV than low-grade counterparts. Differently, AEF characterizes the dynamic arterial perfusion status of tumors, directly mirroring the degree of abnormal angiogenesis induced by HIF-VEGF pathway activation (9). Higher AEF indicates richer arterial blood supply and more active tumor proliferation, which is another core pathological feature of high-grade ccRCC. Compared with static conventional morphological features (tumor necrosis, calcification, margin), ECV and AEF provide continuous, quantitative, and mechanism-based TME information, capturing the microscopic malignant heterogeneity that cannot be identified by visual evaluation alone, thus offering robust incremental predictive value for tumor grading. Notably, tumor diameter, AEF, and HCT showed significant inter-group differences in the internal cohort but lost statistical significance in the external validation cohort, with only ECV remaining discriminative. This finding reflects inherent inter-institutional heterogeneity in patient case-mix and CT acquisition conditions. Specifically, differences between the two cohorts in patient demographics, CT scanning parameters (e.g., delay time, contrast protocol), and the time interval between blood sampling and CT acquisition may have attenuated the predictive performance of these markers. This further highlights the advantage of ECV as a robust cross-institutional imaging biomarker, while other markers require cautious application with institution-specific calibration.

Machine learning methods are increasingly applied in medical imaging and radiomics due to their ability to integrate multidimensional data and capture nonlinear relationships (16–18). We tested six ML models and found Logistic Regression (LR) was optimal: it avoided overfitting (unlike XGBoost/LightGBM) and maintained high performance in external validation. Importantly, SHAP analysis enhanced model transparency by quantifying feature contributions—addressing a key limitation of “black-box” models and facilitating clinical acceptance. Compared with recently published ML and radiomics models for ccRCC preoperative grading (19–25), our model exhibits distinct advantages in predictive stability, clinical interpretability, and external generalization. Most existing studies rely on high-dimensional artificial radiomic texture features or black-box deep learning architectures, which lack clear biological implications and are prone to overfitting, resulting in declined external validation performance with external AUC mostly ranging from 0.74 to 0.82 and relatively low specificity of 75%–88%. Several latest CT-based radiomics models achieved favorable external AUC up to 0.912, but they were constructed based on numerous uninterpretable high-throughput features without validated quantitative imaging biomarkers, limiting clinical translation. Multiparametric MRI-based grading models also achieved promising predictive accuracy, yet they require complex multi-sequence acquisition and are limited by single-center validation, restricting widespread clinical application. By comparison, our optimized logistic regression model achieves balanced and reliable multicenter performance, with a competitive external validation AUC of 0.857 and a notably high specificity of 92.9%, which effectively reduces false-positive predictions and avoids over-treatment for low-grade ccRCC patients. More importantly, distinct from pure radiomic models, our model incorporates biologically interpretable quantitative imaging markers, among which ECV represents the most stable cross-center biomarker, whereas other predictors exhibited inconsistent performance across cohorts. SHAP-based interpretability analysis mitigates the “black-box” limitation of conventional artificial-intelligence models and improves clinical credibility. Clinically, our interpretable ML model enables noninvasive, preoperative individualized risk stratification for ccRCC. Preoperative identification of high-grade ccRCC can assist clinicians in formulating aggressive surgical strategies and arranging intensive postoperative follow-up to prevent tumor recurrence and metastasis. MVD s, nephron-sparing surgery should be prioritized. Active surveillance may only be considered for carefully selected subgroups such as small-sized tumors or patients with high surgical risk after multidisciplinary assessment, consistent with EAU and NCCN guidelines.

Several limitations should be acknowledged. First, this was a retrospective study, and potential selection bias cannot be excluded. Second, although an independent external cohort was included, the sample size remained limited, and multicenter validation with larger cohorts is warranted. In addition, sex distribution differed significantly between low- and high-grade subgroups in the external validation cohort (P = 0.022), which was not observed in the internal dataset. Such inter-center demographic heterogeneity may stem from institution-specific patient case spectra. Although sex was not identified as an important predictor by SHAP analysis, this baseline discrepancy could potentially influence external-validation performance and further underscores the necessity of large-scale multicenter validation with well-balanced patient subgroups. Third, we applied the SMOTE technique to mitigate class imbalance within the training cohort. Synthetic oversampling may inflate training-set predictive performance and yield over-optimistic AUC values. Therefore, model generalizability should be interpreted based on internal and external validation results rather than training-dataset metrics alone. Furthermore, inter-institutional baseline heterogeneity existed in the external test cohort; several clinical and imaging variables that were discriminative in the internal cohort lost their statistical significance in external patients. Such population-level case-mix differences may challenge model generalizability. Although ECV remained the dominant predictive feature and the model achieved acceptable overall external performance, these heterogeneities highlight the need for further validation in larger and more diverse patient populations. Fourth, the model exhibited high specificity but suboptimal sensitivity (60.9%) in the external test cohort, leading to a considerable false-negative rate for high-grade ccRCC. This carries clinical risks of under-stratifying patients who require aggressive treatment. The present cut-off value prioritizes high specificity to reduce false-positive predictions, which comes at the cost of reduced sensitivity. Alternative thresholds with higher sensitivity may be selected for scenario-specific clinical use at the expense of specificity. Accordingly, our model should serve only as an adjunctive auxiliary risk-stratification tool instead of a definitive diagnostic criterion. Comprehensive clinical and radiological evaluation remains mandatory regardless of model output. Fifth, ECV calculation depends on hematocrit (HCT) obtained from blood tests within 24 hours prior to CT acquisition. Acute HCT fluctuations induced by clinical interventions such as intravenous hydration may alter computed ECV values. We excluded patients receiving dialysis; however, complete documentation of intravenous hydration status was not uniformly available in this retrospective cohort, which represents a potential confounder. Future prospective studies should enforce standardized patient clinical conditions and strict laboratory-imaging time windows. Sixth, we adopted single-slice 2D ROIs instead of volumetric whole-tumor segmentation, which may introduce sampling bias and incompletely reflect intratumoral spatial heterogeneity; volumetric whole-tumor ROI strategies should be prioritized in future prospective investigations. Although basic voxel resampling and HU normalization were implemented for image preprocessing, we did not apply dedicated cross-center batch-effect correction such as ComBat; residual scanner-related batch effects may persist and affect cross-site model performance. Further validation of inter-scanner and inter-reader reproducibility of ECV measurements is needed for real-world translation. Finally, ECV measurement depends on CT acquisition protocols and post-processing procedures … Future prospective studies should enforce standardized patient clinical conditions, strict laboratory-imaging time windows, and complete documentation of intravenous hydration-related confounders. Finally, ECV measurement depends on CT acquisition protocols and post-processing procedures, which may affect reproducibility across different institutions and scanners. While traditional machine learning algorithms were adopted in this study for their interpretability, deep learning and multimodal fusion approaches may provide further improvements in predictive performance and should be investigated in future work. Future studies should focus on several directions: (1) conducting large-scale, multicenter, prospective studies to validate the generalizability of our findings; (2) exploring the integration of multiparametric imaging modalities such as MRI or PET-CT with CT-derived ECV; (3) combining imaging-based features with genomic or molecular biomarkers to establish a more comprehensive predictive framework; and (4) developing clinically deployable, real-time decision support tools that can be integrated into routine workflows to facilitate precision medicine in ccRCC management.

## 5 Conclusion

This study demonstrated that an interpretable machine learning model incorporating CT-derived ECV, clinical, and imaging features can effectively predict the WHO/ISUP grading of ccRCC preoperatively. The model exhibited good performance and interpretability, offering a promising noninvasive tool for individualized clinical decision-making. Further large-scale, multicenter studies are required to validate and extend these findings, paving the way toward clinical implementation.

## Data Availability

The raw data supporting the conclusions of this article will be made available by the authors, without undue reservation.

## References

[1] Siegel RL Miller KD Wagle NS Jemal A . Cancer statistics, 2023. CA Cancer J Clin. (2023) 73:17–48. doi: 10.3322/caac.21763 36633525

[2] Jonasch E Walker CL Rathmell WK . Clear cell renal cell carcinoma ontogeny and mechanisms of lethality. Nat Rev Nephrol. (2021) 17:245–61. doi: 10.1038/s41581-020-00359-2 33144689 PMC8172121

[3] Delahunt B Eble JN Egevad L Samaratunga H . Grading of renal cell carcinoma. Histopathology. (2019) 74:4–17. doi: 10.1111/his.13735 30565310

[4] Chen Z Zhu H Shu H Zhang J Gu K Yao W . Preoperative prediction of WHO/ISUP grade of ccRCC using intratumoral and peritumoral habitat imaging: multicenter study. Cancer Imaging. (2025) 25:59. doi: 10.1186/s40644-025-00875-z 40319322 PMC12049773

[5] Nishimuta Y Tsurumaru D Kai S Maehara J Asayama Y Oki E . Extracellular volume fraction determined by equilibrium contrast-enhanced computed tomography: correlation with histopathological findings in gastric cancer. Jpn J Radiol. (2023) 41:752–9. doi: 10.1007/s11604-023-01393-3 36735208 PMC10313564

[6] Chen Y Jiang J Yan C Jiang J Shi B Xu Z . Prediction of tumor regression grade in far-advanced gastric cancer after preoperative immuno-chemotherapy using dual-energy CT-derived extracellular volume fraction. Eur Radiol. (2025) 35:93–104. doi: 10.1007/s00330-024-10737-0 38981889

[7] Liu J Zhang X Lv R Zhang X Wang R Zeng X . Predictive value of extracellular volume fraction determined using enhanced computed tomography for pathological grading of clear cell renal cell carcinoma: a preliminary study. Cancer Imaging. (2025) 25:49. doi: 10.1186/s40644-025-00866-0 40186299 PMC11969730

[8] Avanzo M Wei L Stancanello J Vallières M Rao A Morin O . Machine and deep learning methods for radiomics. Med Phys. (2020) 47:e185–202. doi: 10.1002/mp.13678 32418336 PMC8965689

[9] Currie G Hawk KE Rohren E Vial A Klein R . Machine learning and deep learning in medical imaging: Intelligent imaging. J Med Imaging Radiat Sci. (2019) 50:477–87. doi: 10.1016/j.jmir.2019.09.005 31601480

[10] Wei Z Bai X Xv Y Chen SH Yin S Li Y . A radiomics-based interpretable machine learning model to predict the HER2 status in bladder cancer: a multicenter study. Insights Imaging. (2024) 15:262. doi: 10.1186/s13244-024-01840-3 39466475 PMC11519251

[11] Moch H . The WHO/ISUP grading system for renal carcinoma. Pathologe. (2016) 37:355–60. doi: 10.1007/s00292-016-0171-y 27271258

[12] Takumi K Nagano H Myogasako T Nakano T Fukukura Y Ueda K . Feasibility of iodine concentration and extracellular volume fraction measurement derived from the equilibrium phase dual-energy CT for differentiating thymic epithelial tumors. Jpn J Radiol. (2023) 41:45–53. doi: 10.1007/s11604-022-01331-9 36029365 PMC9813095

[13] Adams LC Jurmeister P Ralla B Bressem KK Fahlenkamp UL Engel G . Assessment of the extracellular volume fraction for the grading of clear cell renal cell carcinoma: first results and histopathological findings. Eur Radiol. (2019) 29:5832–43. doi: 10.1007/s00330-019-06087-x 30887194

[14] Ming S Zhang W Wang M Li J Shi D Wang B . Contrast-enhanced CT-derived extracellular volume fraction predicts survival in postoperative pancreatic cancer patients. Eur J Radiol. (2025) 191:112273. doi: 10.1016/j.ejrad.2025.112273 40618544

[15] Luo Y Liu L Liu D Shen H Wang X Fan C . Extracellular volume fraction determined by equilibrium contrast-enhanced CT for the prediction of the pathological complete response to neoadjuvant chemoradiotherapy for locally advanced rectal cancer. Eur Radiol. (2023) 33:4042–51. doi: 10.1007/s00330-022-09307-z 36462046

[16] Adlung L Cohen Y Mor U Elinav E . Machine learning in clinical decision making. Med. (2021) 2:642–65. doi: 10.1016/j.medj.2021.04.006 35590138

[17] Chen M Copley SJ Viola P Lu H Aboagye EO . Radiomics and artificial intelligence for precision medicine in lung cancer treatment. Semin Cancer Biol. (2023) 93:97–113. doi: 10.1016/j.semcancer.2023.05.004 37211292

[18] Xin W Rixin S Linrui L Zhihui Q Long L Yu Z . Machine learning-based radiomics for predicting outcomes in cervical cancer patients undergoing concurrent chemoradiotherapy. Comput Biol Med. (2024) 177:108593. doi: 10.1016/j.compbiomed.2024.108593 38801795

[19] Zhou Z Qian X Hu J Ma X Zhou S Dai Y . CT-based peritumoral radiomics signatures for malignancy grading of clear cell renal cell carcinoma. Abdom Radiol (NY). (2021) 46:2690–8. doi: 10.1007/s00261-020-02890-z 33427908

[20] Li Y Lin M Sun L Dong C Yang J Li X . An explainable clinical-radiomics machine learning model for preoperative prediction of WHO/ISUP nuclear grade in clear cell renal cell carcinoma. Abdom Radiol. (2026). doi: 10.1007/s00261-026-05521-1 42033642 PMC13672578

[21] Li X Song S Ge W Wang H Liu W Qi X . Application of contrast-enhanced CT-based radiomics with machine learning to predict WHO/ISUP grading in clear cell renal cell carcinoma. BMC Cancer. (2026) 26:636. doi: 10.1186/s12885-026-15794-w 41928166 PMC13188344

[22] Tao X Shan S Chen X Yu Z Qi H . An interpretable machine learning model based on habitat radiomics combined with deep learning for predicting the WHO/ISUP grade of patients with clear cell renal cell carcinoma. BMC Med Imaging. (2026) 26:221. doi: 10.1186/s12880-026-02285-4 41864916 PMC13130805

[23] Yang D Mei H Jiao P Zheng Q . Biological interpretable machine learning model for predicting pathological grading in clear cell renal cell carcinoma based on CT urography peritumoral radiomics features. Bioengineering-Basel. (2025) 12:1125. doi: 10.3390/bioengineering12101125 41155123 PMC12561653

[24] Yang Y Zhang Z Zhang H Liu M Zhang J . Machine learning-based multiparametric MRI radiomics nomogram for predicting WHO/ISUP nuclear grading of clear cell renal cell carcinoma. Front Oncol. (2024) 14:1467775. doi: 10.3389/fonc.2024.1467775 39575426 PMC11578869

[25] Li S Zhou Z Gao M Liao Z He K Qu W . Incremental value of automatically segmented perirenal adipose tissue for pathological grading of clear cell renal cell carcinoma: a multicenter cohort study. Int J Surg. (2024) 110:4221–30. doi: 10.1097/JS9.0000000000001358 38573065 PMC11254242

