# Supplementary material for: Interpretable machine learning for preoperative prediction of WHO/ISUP grade in clear cell renal cell carcinoma using contrast-enhanced CT–derived extracellular volume fraction
Source: Front Oncol. 2026 Oct 13;16:1851636. doi: 10.3389/fonc.2026.1851636 (PMC13710075; doi:10.3389/fonc.2026.1851636)
Supplement: Supplementary file 1 [file Table1.docx]

Table S1 All baseline characteristics between the training and internal validation cohort

|  | Training cohort  (n=128) | Validation cohort  (n=56 ) | *P value* |
| --- | --- | --- | --- |
| Age | 59.73±11.08 | 58.95±9.69 | 0.646 |
| Sex |  |  | 0.320 |
| Female | 44 | 24 |  |
| Male | 84 | 32 |  |
| Diameter | 5.30±2.49 | 5.77±1.99 | 0.210 |
| AEF | 1.44±0.28 | 1.44±0.23 | 0.969 |
| ECV | 0.50±0.12 | 0.51±0.12 | 0.700 |
| Necrosis |  |  | 0.492 |
| None | 20 | 6 |  |
| Present | 108 | 50 |  |
| Calcification |  |  | 1.000 |
| None | 114 | 50 |  |
| Present | 14 | 6 |  |
| Margin |  |  | 1.000 |
| Clear | 93 | 41 |  |
| Blurry | 35 | 15 |  |
| Intratumoral vessels |  |  | 0.862 |
| None | 89 | 38 |  |
| Present | 40 | 16 |  |
| Renal vein tumor thrombus |  |  | 0.725 |
| None | 121 | 54 |  |
| Present | 7 | 2 |  |
| Renal collecting system invasion |  |  | 0.813 |
| None | 112 | 16 |  |
| Present | 48 | 8 |  |
| Lymph node metastasis |  |  | 1.000 |
| None | 125 | 55 |  |
| Present | 3 | 1 |  |
| Enhancement pattern |  |  | 0.586 |
| Homogeneous | 126 | 54 |  |
| Heterogeneous | 2 | 2 |  |
| WHO/ISUP grades |  |  | 1.000 |
| Low | 80 | 48 |  |
| High | 35 | 21 |  |

Abbreviations:  AEF: Arterial enhancement fraction; ECV: Extracellular volume fraction.
