# Supplementary material for: Interpretable machine learning for preoperative prediction of WHO/ISUP grade in clear cell renal cell carcinoma using contrast-enhanced CT–derived extracellular volume fraction
Source: Front Oncol. 2026 Oct 13;16:1851636. doi: 10.3389/fonc.2026.1851636 (PMC13710075; doi:10.3389/fonc.2026.1851636)
Supplement: Supplementary file 2 [file Table2.docx]

Table S2 All parameter collinearity diagnostic analysis

|  | B | t *value* | *P value* | VIF |
| --- | --- | --- | --- | --- |
| Age | 0.002 | 0.915 | 0.361 | 1.087 |
| Sex | 0.124 | 1.969 | 0.051 | 1.256 |
| HCT | -1.042 | -1.779 | 0.077 | 1.372 |
| Diameter | 0.017 | 0.948 | 0.345 | 2.347 |
| AEF | 0.362 | 3.226 | 0.002 | 1.167 |
| ECV | 1.977 | 7.641 | 0.001 | 1.227 |
| Necrosis | -0.109 | -1.283 | 0.201 | 1.194 |
| Calcification | 0.050 | 0.526 | 0.600 | 1.194 |
| Margin | 0.100 | 1.300 | 0.195 | 1.600 |
| Intratumoral vessels | 0.166 | 2.155 | 0.033 | 1.694 |
| Renal vein tumor thrombus | 0.419 | 2.710 | 0.007 | 1.513 |
| Renal collecting system invasion | -0.105 | -0.945 | 0.346 | 1.890 |
| Lymph node metastasis | 0.178 | 0.879 | 0.380 | 1.178 |
| Enhancement pattern | -0.168 | -0.876 | 0.382 | 1.056 |

Abbreviations: VIF:variance inflation factor; HCT: Hematocr ; AEF: Arterial enhancement fraction; ECV: Extracellular volume fraction.
